# Pathological Dentition

**Published:** 1873-12

**Authors:** James W. White


### ARTICLE VII.

### *Pathological Dentition.*

BY JAMES W. WHITE, M. D.

Dentition, though a physiological process, is nevertheless recognized as a frequent cause of constitutional disturbance. Doubtless there are extremists who overestimate the average influence of this process as a disturbing element, as there are those who underrate the difficulties which may attend it. Pathological dentition is by many considered a secondary affection,—a single link in a chain of deranged actions,—and, even when a little patient indicates unmistakably the local irritation, relief is sought by general medication,—relaxants, derivatives, calmatives, febrifuges, etc.; then by local emollients, fomentations and anodynes; and lastly, if at all, by lancing the gums, when redness, tumefaction, induration, or the whiteness of the coming tooth seems to demand it. These signs are indeed assumed to be the only possible justification of the operation. If the gums are tumid, tense, and shining, swollen up into a kind of little tumor over a particular tooth; if an unhealthy ulceration with a sloughy appearance forms upon the summit of the gum; then, say our text-books and writers upon the diseases of infancy,—*then* we may sometimes resort to incision of the gum.

"In forming a diagnosis," says one of the highest authorities, "whether a disease present during the time of teething is consequent upon some derangement of this process, or upon an abnormal condition of some other organ or organs of which the dental difficulty is but itself a symptom, the state of the jaws must be the principal guide. If in the presence of symptoms which might arise from teething, we find that the teeth are not pressing forward towards the surface of the gums, and that the latter maintain their normal appearance, it will be useless to have recourse to the gum lancet." Young practitioners are cautioned, by a recent writer, not to display their ignorance by the use of the lancet, except the *local* indications imperatively demand it. The local signs, it is to be inferred, are tumefaction, redness, induration, ulceration, and the whiteness of the presenting tooth. The direct pressure upon the fibrous tissue is thus assumed to be the cause of the various and serious complications which are too frequently associated with the period of the primary dentition. It is doubtless true that a hyperæmic condition of the gums may be caused by the growth or eruption of the teeth proceeding more rapidly than does the absorption of their tegumental covering, and that the undue pressure thus caused may occasion trouble, by the irritation of the nerves of the gum tissues,—manifested locally by tumefaction, soreness, redr ess, or ulceration; systematically, by fever, irritability, sleeplessness, etc. It is also admitted that judicious treatment of pathological dentition should in all cases include hygienic care, and that constitutional medical, as well as local surgical, interference is generally demanded. Nor is it claimed that in the perversion of this physiological process is to be found an explanation of all the ills to which human infancy is heir; but we assume that pain so intense and unremitting as to destroy the appetite for food, to cause wakefulness, irritability, thirst, fever, diarrhœa or constipation, congestion, convulsions, and death, may be due to the irritation of dentition *without the existence of a single local indicatian.* In other words, that the most serious complications of dentition are not caused by the pressure of the advancing tooth upon the gums, but by the backward pressure of the resisting gums upon the developing and sensitive *pulp*, giving rise to a true toothache, comparable only to that exquisite torture which is experienced in after-life from an exposed and irritated plup.

If such a condition of things is possible, it will readily be seen that there can be no question as to the extent of the mischief which may result. The association of the fifth pair of nerves, which supplies the dental filaments, with the great sympathetic so connects the teeth with the entire economy that the pathological bearings of such deranged action may not be limited. That such a condition may exist will be readily understood, if it is remembered that at the period of eruption the roots of the teeth are as yet incomplete; that instead of the conical termination and minute foramen which characterize perfected teeth, the aperture is quite large, and its edges thin and sharp. In estimating, therefore, the amount of constitutional disturbance which may result because of a want of accordance between the eruption of a tooth and the absorption of the superimposed tissues which impede it, we may imagine the sensitive pulp, made up of arteries' veins, and nerves, in a condition of irritation from augmented vascular and nervous action,—a morbid activity of the process of dentition,—followed by determination, stasis, and congestion producing a hyperæmia sufficient to cause the protrusion of the mass from the in. complete aperture of the root; which, being impressed upon by its thin, sharp edges, is sufficient cause for any amount of constitutional disturbance of which it is possible to conceive.

Under such circumstances it is not difficult to comprehend the inefficacy of any or all hygienic measures; of relaxants and febrifuges; of local emollients and anodynes. It is also easy to understand how the thorough lancing of the gums, over the tooth or teeth thus situated, may, by removal of the pressure, give a relief so immediate and complete that there shall be no room for doubt as to the correctness of the diagnosis.

The *general* indications of what may not inaptly be termed infantile odontalgia are precisely what might *a priori* be expected. The child, at first simply uneasy, becomes by rapid stages fretful, troublesome, peevish, cross, vindictive ; cries persistently, or stops crying only to scream ; or, if quiet for an instant, will be found to have its thumb or fingers thrust between the jaws, the chewing upon which seems to afford a momentary cessation of anguish, but only momentary. It refuses food, throws down its toys if handed to it, as though in a passion, and is outraged by any attempt to amuse it. To these persistent unmistakable evidences of irritability are added a flushed face, corrugated brow, compressed lip, intolerance of light, and disturbed, broken sleep, the desire and effort to sleep seeming to be thwarted by fresh accessions of pain, until the little one sinks exhausted into a troubled slumber, but of short duration. Concomitant with these manifestations, or quickly succeeding them, will be some of the various systemic complications, too frequently with fatal ending, and still *no local indication* of the trouble which is consuming the young life.

A case recently under the care of the writer afforded a marked confirmation of these views. A child one year of age, with the four superior and two inferior incisors in position, after three weeks of restlessness, wakefulness, loss of appetite, fever, paroxysms of pain, and rapid emaciation,—all without obvious cause, certainly without the slightest local indications of trouble in the mouth,—was *cured* by free crucial incisions over the molar teeth, the improvement being so evidently the result of the operation that the relation of cause and effect was plainly recognized by every member of the family.

Such cases are not exceptional, and suggest a more careful investigation of the developmental processes of dentition in otherwise unexplainable diseases of infancy.—*Medical Times.*

